# Functional Connectivity Lateralisation Shift of Resting State Networks is Linked to Visuospatial Memory and White Matter Microstructure in Relapsing–Remitting Multiple Sclerosis

**DOI:** 10.1007/s10548-021-00881-x

**Published:** 2021-11-22

**Authors:** Dániel Veréb, Márton Attila Kovács, Krisztián Kocsis, Eszter Tóth, Bence Bozsik, András Király, Bálint Kincses, Péter Faragó, Zsanett Fricska-Nagy, Krisztina Bencsik, Péter Klivényi, Zsigmond Tamás Kincses, Nikoletta Szabó

**Affiliations:** 1 Department of Radiology, Albert Szent-Györgyi Clinical Center, University of Szeged, Szeged, Hungary; 2 Department of Neurobiology, Care Sciences and Society, Karolinska Institutet, Stockholm, Sweden; 3 Department of Neurology, Albert Szent-Györgyi Clinical Center, University of Szeged, Szeged, Hungary; 4 Department of Psychiatry, Albert Szent-Györgyi Clinical Center, University of Szeged, Szeged, Hungary; 5 Institute of Diagnostic and Interventional Radiology and Neuroradiology, University Hospital Essen, Essen, Germany; 6 Neuroimaging Research Group, Department of Radiology, Albert Szent-Györgyi Clinical Center, University of Szeged, Szeged, Semmelweis u. 6, 6725 Hungary

**Keywords:** Multiple sclerosis, Functional MRI, Diffusion tensor imaging, Lateralisation

## Abstract

Laterality patterns of resting state networks (RSN) change in various neuropsychiatric conditions. Multiple sclerosis (MS) causes neuro-cognitive symptoms involving dysfunctional large-scale brain networks. Yet, whether healthy laterality patterns of RSNs are maintained in MS and whether altered laterality patterns explain disease symptoms has not been explicitly investigated. We analysed functional MRI and diffusion tensor imaging data from 24 relapsing–remitting MS patients and 25 healthy participants. We performed group-level independent component analysis and used dual regression to estimate individual versions of well-established RSNs. Voxelwise laterality indices were calculated for each RSN. Group differences were assessed via a general linear model-based approach. The relationship between functional laterality and white matter microstructural asymmetry was assessed using Tract-Based Spatial Statistics. Spearman’s correlation was calculated between laterality indices and Brief International Cognitive Assessment for Multiple Sclerosis scores. Functional laterality of the dorsal attention network showed a significant leftward shift in the MS group in the posterior intraparietal sulcus (p < 0.033). Default-mode network laterality showed a significant leftward shift in the MS group in the angular gyrus (p < 0.005). Diminished dorsal attention network laterality was associated with increased fractional anisotropy asymmetry in the superior longitudinal fasciculus (p < 0.02). In the default-mode network, leftward laterality of the angular gyrus was associated with higher BVMT-R scores (R = − 0.52, p < 0.023). Our results confirm previous descriptions of RSN dysfunction in relapsing–remitting MS and show that altered functional connectivity lateralisation patterns of RSNs might contibute to cognitive performance and structural remodellation even in patients with mild clinical symptoms.

## Introduction

The macro- and microstructural damage of both the white and grey matter in multiple sclerosis (MS) lead to the dysfunction of large scale resting state neural networks, which has been linked to worsening cognitive performance, an increasingly recognised disease feature present in 43–70% of the patient population (Chiaravalloti and DeLuca 2008). A major part of neuroimaging studies in MS focused on altered connectivity in resting state networks (RSNs), collections of brain regions exhibiting synchronous activity during rest (Smith et al. 2009). Although MS-related RSN dysfunction has been investigated thoroughly (Rocca et al. 2018; Sbardella et al. 2015; Zhou et al. 2014), several features remain to be characterised that might add to our understanding of how cognitive dysfunction develops in MS patients. For example, functional lateralisation is an important characteristic of the brain thought to have evolutional advantages in supporting higher cognitive function (Gerrits et al. 2020; Petit et al. 2015; Vallortigara 2006; Vallortigara et al. 1999). Accordingly, RSNs appear lateralised in healthy subjects, the lateralisation pattern changing with age and gender (Agcaoglu et al. 2015; Cai et al. 2019), while also bearing relevance to cognitive ability (Gotts et al. 2013). Altered lateralisation patterns contribute to impaired cognitive performance across several domains in neuropsychiatric disorders, e.g. schizophrenia (Ribolsi et al. 2014) or autism (Floris et al. 2016), and appear in mild cognitive impairment and Alzheimer’s disease as well (Liu et al. 2018). One of the main domains where lateralisation is particularly pronounced and its erosion signals early cognitive deterioration is spatial attention, subserved by a right hemisphere dominant neural system (Corbetta and Shulman 2002). Attention deficits develop early on in MS patients and are heavily interwoven with decreased information processing speed (Roth et al. 2015) and other well-established markers of cognitive dysfunction in MS (Langdon et al. 2012). Additionally, both verbal and spatial working memory are often implicated in MS as part of the typical profile of cognitive impairment (Chiaravalloti and DeLuca 2008). These functions also exhibit a lateralised underlying functional architecture characterised by side preference in activation studies (Reuter-Lorenz et al. 2000). The involvement of these functions fits the characteristic pattern of cognitive impairment that results from extensive, diffuse damage to the white matter (Filley 2012). As cognitive impairment develops, (mal)adaptive changes take place in resting state networks (Filley and Fields 2016; Tahedl et al. 2018a). Since these alterations manifest in the redistribution of connectivity in resting state networks, it is likely that a shift in hemispheric dominance also occurs. This would bring about altered lateralisation patterns in functional connectivity that might play a part in MS-related neurocognitive symptoms. A lateralisation of both structural and functional changes compared to healthy participants has been described in previous studies, where changes in the right hemisphere were more discriminative for MS (see e.g. (Tahedl et al. 2018b) for a review). However, it has not been explicitly investigated whether the lateralised pattern of RSNs characteristic of the healthy brain is preserved in MS, especially in patients with mild disability. Exploring changes in functional lateralisation patterns can be important as functional network adaptation relates closely to cognitive performance (Helekar et al. 2010). The identification of common patterns allows for their exploitation as objective markers measuring the efficacy of intervention in cognitive rehabilitation strategies (Tomassini et al. 2012). In this study, we use resting state fMRI data to investigate how functional connectivity within RSNs is lateralised in MS patients compared to a healthy control group. We then examine whether deviation from the healthy lateralisation pattern corresponds to the patients’ cognitive performance, as measured by the widely used Brief International Cognitive Assessment for Multiple Sclerosis (BICAMS) screening battery (Langdon et al. 2012). Furthermore, we assess whether the asymmetry of white matter microstructure corresponds to changes in functional lateralisation.

Methods.

### Participants

MRI data from 24 patients with relapsing–remitting multiple sclerosis and 25 healthy participants were analysed in this study. Patients were recruited from the Multiple Sclerosis Outpatient Clinic at the Department of Neurology, University of Szeged. Healthy participants were colleagues and family members of the patients. At the time of the scan, all MS patients were on disease-modifying treatment and had no relapses at least 3 months prior to or after the scan. All participants were right-handed. Healthy controls had no neurological or psychiatric conditions and MS patients had no other neuropsychiatric conditions apart from MS. Written informed consent was obtained from all participants, and the local ethics committee approved the study (Ref No. 35/2017).

### Cognitive Tests

We measured cognitive function in the MS group using the Hungarian validated version of the BICAMS battery (Sandi et al. 2015). This collection of tests includes three subtests: the Symbol Digit Modalities Test (SDMT) for assessing information processing speed, the Brief Visuospatial Memory Test Revised (BVMT-R) for assessing visuospatial working memory and the immediate recall subtest of the California Verbal Learning Test 2 for assessing verbal working memory (CVLT-II). Patients completed the BICAMS battery on the day of the MRI measurements.

### Image Acquisition

Measurements took place on a 3 T GE MR750W Discovery scanner (GE, Milwaukee, USA). 3D T1-weighted structural images using the FSPGR-IR sequence (TR: 5.3 ms TE: 2.1 TI: 450 ms, slice thickness: 1 mm, matrix: 512 × 512, FOV 256 × 256 mm, Slice No. 312, whole brain coverage, flip angle: 12°) and T2*-weighted BOLD EPI images (TR: 2500 ms, TE: 27 ms, 44 × 3 mm axial slices providing whole-brain coverage, in-plane resolution: 3 mm × 3 mm, FOV: 288mmx288mm, matrix 96 × 96, flip angle: 81°, interleaved acquisition scheme) were obtained for all participants. For the MS group, 30-directional diffusion-weighted images with three non-diffusion weighted volumes for reference were acquired (TR: 8200 ms, TE: 85 ms, matrix: 96 × 96, flip angle: 90°, in-plane resolution: 2.4 mm x 2.4 mm, FOV: 230mmx230mm, slice thickness: 2.4 mm, 56 axial slices, b: 1000 s/mm^2^).

### Functional MRI Preprocessing

Preprocessing steps were performed via FEAT v6.0.0 as contained in the FMRIB Software Library [FSL, v5.0.10, (Smith et al. 2004)]. Rigid-body realignment of functional scans was performed with MCFLIRT for motion correction, followed by the removal of non-brain tissue using FSL BET (Smith 2002). Resulting volumes underwent slice-timing correction and spatial smoothing with a Gaussian kernel of 6 mm full-width-at-half-maximum. Further motion correction was applied using ICA-AROMA (Pruim et al. 2015), and signal from the white matter and cerebrospinal fluid was removed with nuisance regression. Functional scans then underwent temporal filtering using a high pass filter with a cutoff of 0.01 Hz. Prior to registration, lesion filling was performed on the structural scans of MS patients using the *lesion_filling* tool included in FSL to improve registration accuracy (Battaglini et al. 2012). Lesion masks were drawn manually using the patients’ clinical FLAIR scans and were supervised by an experienced neuroradiologist (ZTK). Functional scans were aligned to structural scans using a boundary-based registration process, and then were further transformed to standard 2 mm MNI-space using non-linear registration with FSL FNIRT.

### Group Independent Component Analysis (ICA)

Since MS-related deficits of resting state networks are widely described in the literature, we performed group-level independent component analysis (ICA) in the healthy cohort to acquire group-average maps of resting state networks using temporal concatenation ICA as implemented in FSL MELODIC (Beckmann and Smith 2004). The number of components was set to 20 to acquire similar network maps as in (Smith et al. 2009). Resulting independent components were classified as resting state network (RSN) or noise based on the spatiotemporal characteristics of the components following recent guidelines (Griffanti et al. 2017). Then we estimated an individual version of each network by projecting the group average spatial map back into each participant’s native space using dual regression (Nickerson et al. 2017).

### Calculation of Voxel Wise Laterality Indices

In order to quantify functional laterality in each participant, we calculated laterality indices using a scheme similar to (Agcaoglu et al. 2015). We warped individual maps of each RSN to a symmetric template (*ICBM 2009c Nonlinear Symmetric* template (Fonov et al. 2009)), then we flipped these maps around the x-axis, and subtracted the flipped RSN map from the unflipped map, acquiring voxelwise laterality indices. To assess group differences, we used a general linear model approach using age and sex as a cofactor since these variables have shown impact on RSN lateralisation (Agcaoglu et al. 2015). Inference was performed voxel wise using a nonparametric permutation test implemented in FSL *randomise*. Symmetric maps created from the group ICA’s probability map (thresholded at p = 0.5) were used as masks during *randomise* in order to isolate homotopic voxels bilaterally contributing to the RSN’s activity. We calculated the partial Spearman’s rank correlation coefficient between laterality indices and BICAMS scores, correcting for age, sex, education, disease duration and treatment regime, as several studies reported differential effects of disease modifying therapies on cognitive disability progression (Landmeyer et al. 2020).

### Preprocessing of Diffusion MRI Data

Diffusion MRI scans first underwent correction for eddy currents using FSL’s *eddy_correct* tool, then were corrected for motion artefacts via a 12 degree-of-freedom affine registration to the first non-diffusion-weighted volume. A diffusion tensor model was fitted in each voxel with FMRIB’s Diffusion Toolkit (v4.0) and scalar descriptors of diffusion properties (fractional anisotropy (FA), mean diffusivity (MD), axial diffusivity (AD), radial/perpendicular diffusivity (RD)) were calculated.

### Relation of Functional Laterality to White Matter Microstructure Lateralisation

To test for possible microstructural correlates of functional laterality alterations, we employed FSL’s *tbss_sym* tool, an extension of the Tract-Based Spatial Statistics (TBSS) method (Smith et al. 2006). During the course of this analysis, the FA images of all subjects were re-aligned into template space via non-linear registration with FSL FNIRT. Next, by averaging FA volumes across subjects, a mean FA volume was calculated, which was thresholded at FA = 0.2 and skeletonised to derive a mean FA skeleton that contains the centre of white matter tracts common to the subject pool. To create a symmetric skeleton that can be used to compare bilateral voxels in the skeleton, the original mean FA volume was left–right flipped and averaged with the original mean FA volume to derive a symmetric mean FA image. The symmetric mean FA volume was fed into the skeletonisation algorithm and all subjects’ pre-aligned FA data were projected to the resulting symmetric skeleton. Symmetrised, skeletonised FA volumes were left–right flipped and subtracted from unflipped volumes to calculate FA asymmetry along major white matter tracts. Since the two sides of these volumes contain identical information (differing by a sign flip only), we kept the right side of the volumes for further analysis, in line with the functional laterality indices described above. Correlation between mean functional laterality indices underlying altered laterality clusters in the MS group and diffusion parameter asymmetry was assessed voxel wise (restricted to previously established structural pathways underlying the given network) in a general linear model framework, using a nonparametric permutation test implemented in FSL *randomise*.

## Results

### Group Independent Component Analysis

**Fig. 1:**
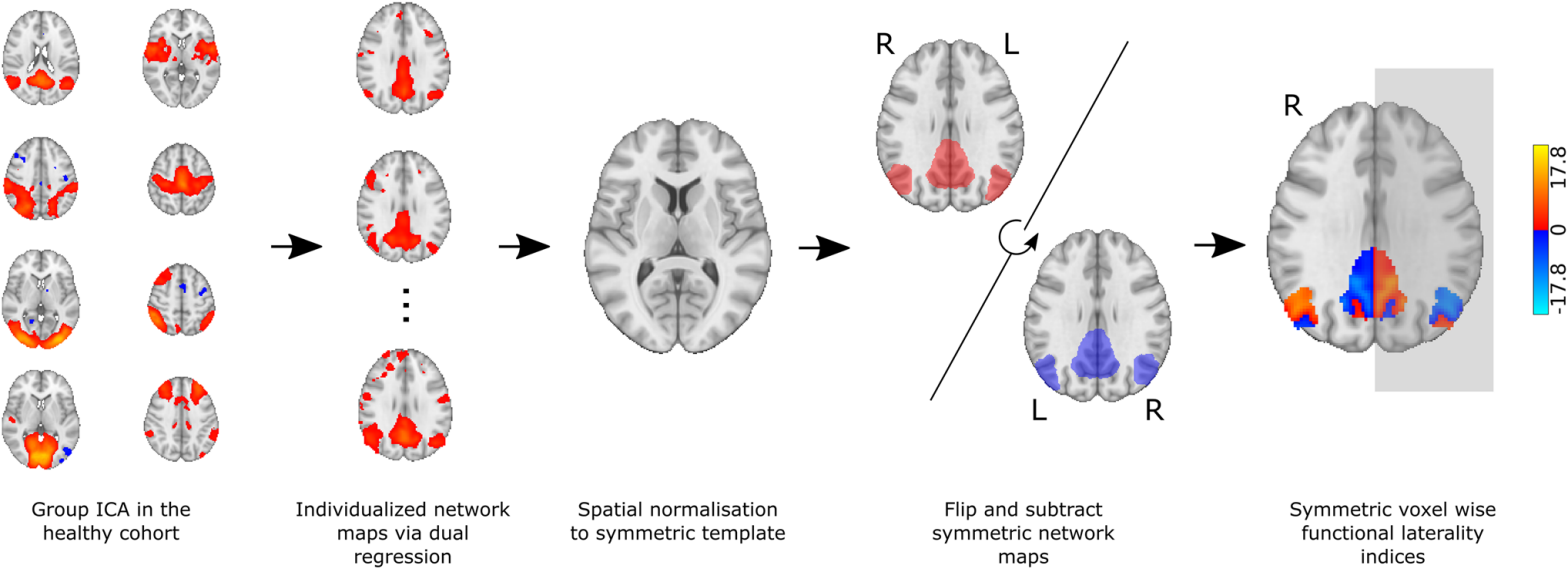
Calculation of functional laterality indices. Following temporal concatenation group ICA in the healthy control group, dual regression was used to estimate individual versions of resting state networks, which were then further normalised to a symmetric template. Symmetric voxelwise laterality index maps were calculated for each subject by subtracting the left–right flipped individualised parameter estimate map from the original. The color bar represents functional laterality indices

**Fig. 2:**
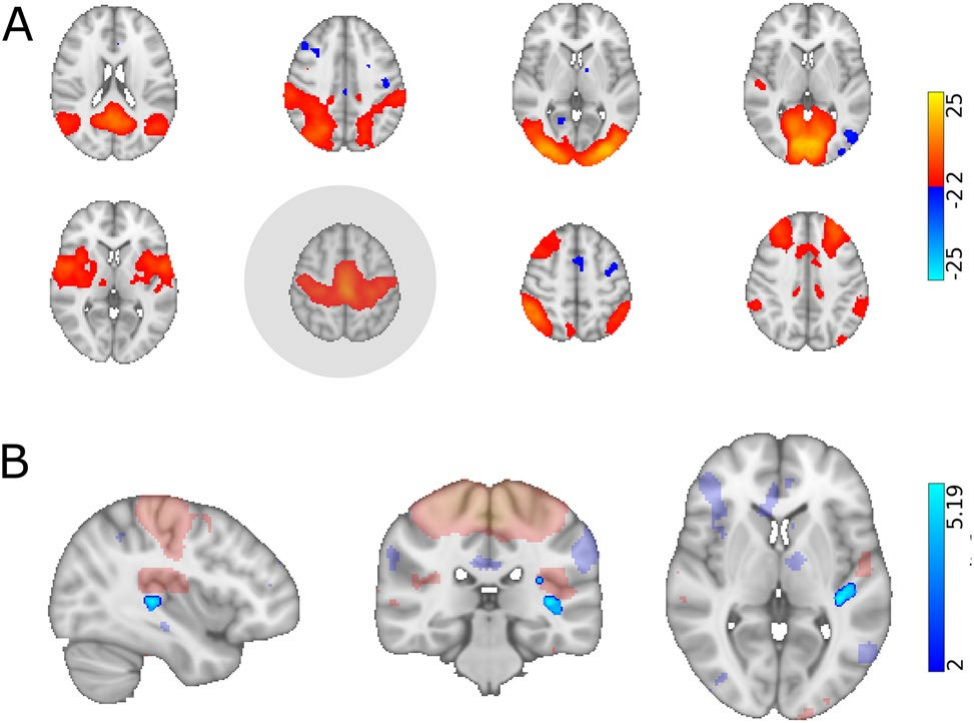
Group independent component analysis and dual regression results. **A** The group ICA analysis produced 8 components resembling previously described resting state networks (from left-to-right): the default mode, dorsal attention, lateral/medial visual, auditory, sensorimotor, frontoparietal and executive networks. Network maps were overlaid on the MNI152 standard template. The color bar depicts Z-values. The sensorimotor network (highlighted in gray) showed reduced connectivity in the MS group (max. voxel MNI coordinates: x = -38 y = -30 z = 4). **B** Altered connectivity of the SMN in the MS group. The SMN spatial map (transparent) and clusters showing significant connectivity differences were overlaid on the MNI152 standard template. The color bar depicts T-statistics

8 components resembling well-described resting state networks were chosen for further analysis, namely: the default mode, dorsal attention, sensorimotor, auditory, medial and lateral visual, executive-control/salience and frontoparietal networks (see Fig. 1 for the spatial maps of these networks). The sensorimotor network showed significantly decreased connectivity in the parietal operculum and near the primary auditory cortex in the MS group (p < 0.013; see Fig. 2).

### Functional Laterality—Group Differences

**Fig. 3:**
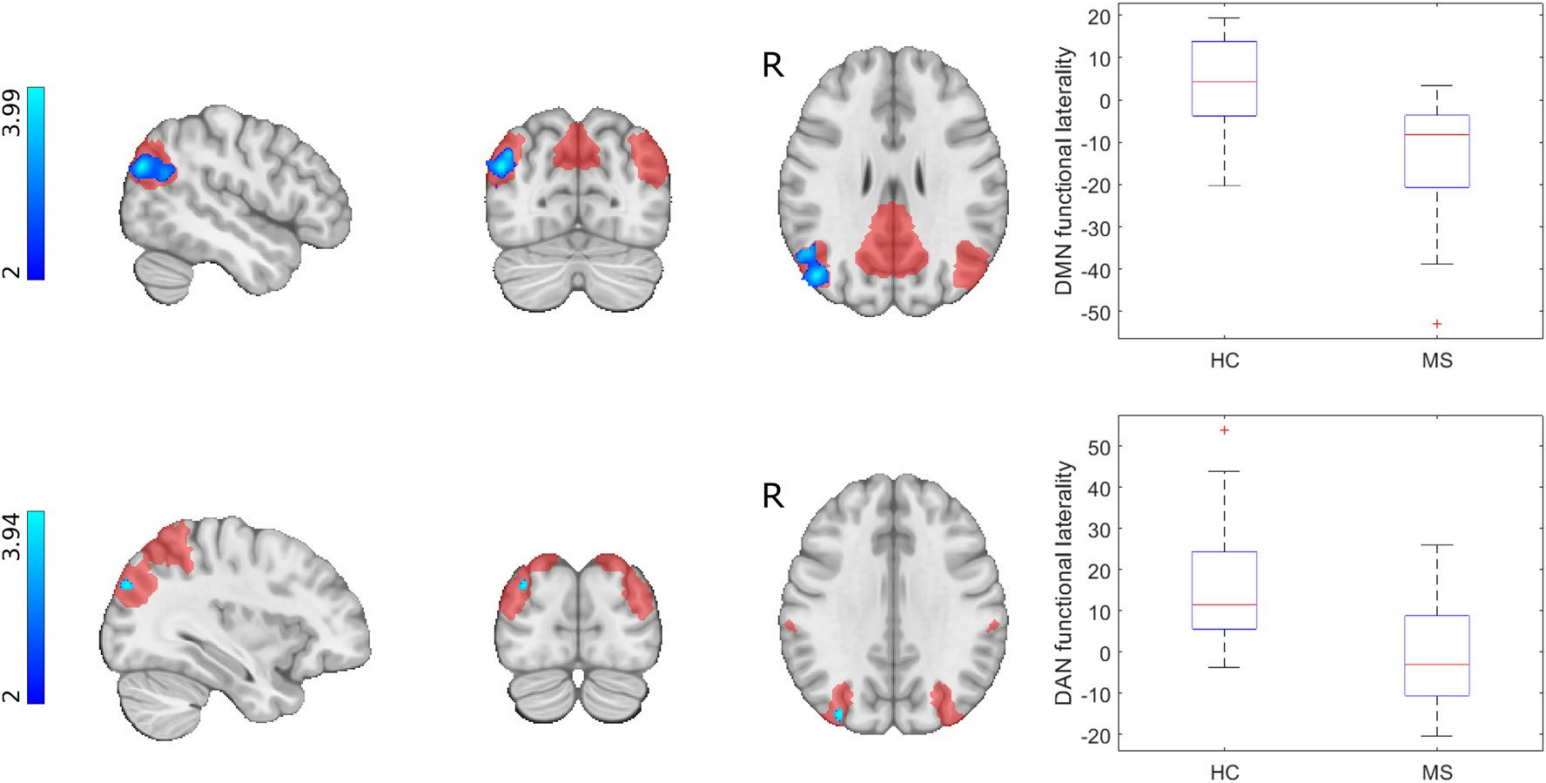
Altered functional laterality in the MS group. Clusters showing significant group differences in functional laterality were superimposed on the symmetric ICBM 2009 template and the symmetrised network masks (depicted in red); blue-light blue clusters show MS < HC (max. voxel MNI coordinates: default mode network – x = 48 y = − 74 z = 28; dorsal attention network – x = 34 y = − 84 z = 32). Color bars depict T-statistics. Boxplots depict mean laterality indices in the two groups that underlie clusters of significant group differences

Functional laterality in the default mode network was significantly decreased in the MS group in the angular gyrus and inferior parietal lobule, indicating a leftward shift in connectivity (p < 0.005, corrected for multiple comparisons). Laterality of the dorsal attention network was significantly diminished in the MS group in the posterior intraparietal sulcus, also indicating a leftward shift of functional laterality (p < 0.033, corrected for multiple comparisons). We subsequently tested whether mean laterality indices underlying the group difference cluster show express lateralisation to the right or left in either group using a two-tailed Wilcoxon signed rank test. The group difference cluster in the default mode network was not significantly lateralized in the healthy group, whereas it showed significant leftward dominance in the MS group (p < 0.001). Laterality of the group difference cluster in the dorsal attention network was significantly right side dominant in the healthy group (p < 0.001) and it did not express significant lateralisation in the MS group. Figure 2 shows the locations of altered laterality (Fig. 3).

### Functional Laterality – Correlation with Cognitive and Clinical Status

Lower laterality indices in the altered region of the default mode network (meaning increased leftward lateralisation) came with significantly higher BVMT-R scores corrected for age, sex, disease duration, education and treatment (R = − 0.52, p < 0.023). Clinical parameters (EDSS, disease duration, lesion volume, number of relapses) did not correlate with laterality indices.

### Functional Laterality—Relationship to Microstructural Asymmetry

**Fig. 4:**
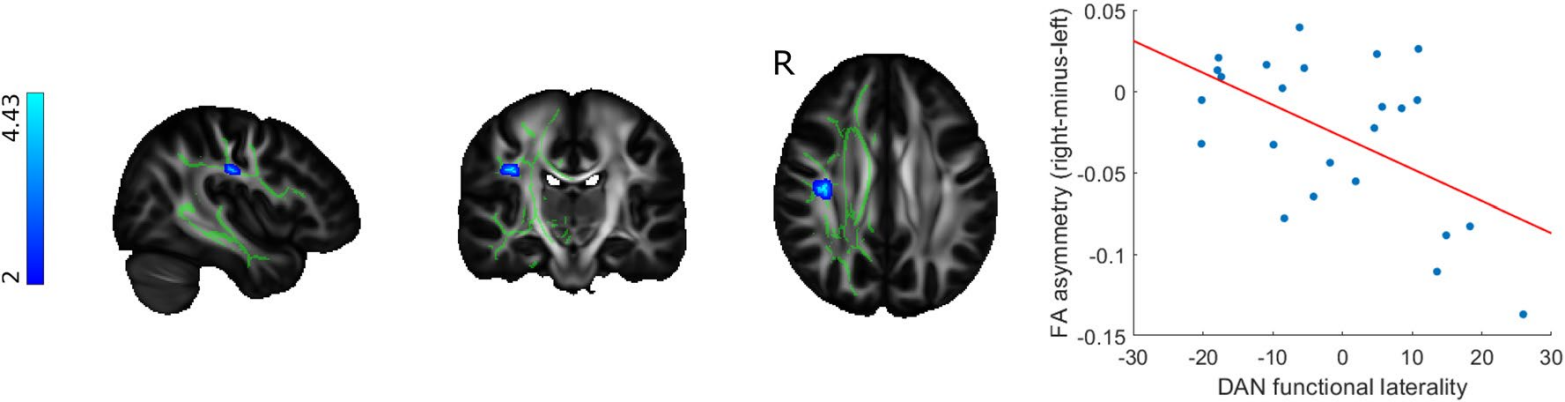
Functional laterality changes in MS patients are connected to white matter microstructural asymmmetry in the dorsal attention network. Clusters showing significant association between dorsal attention network functional laterality and FA asymmetry (shown in blue-light blue) were overlaid on the FSL HCP1065 mean FA template and the symmetrised FA skeleton (depicted in green). The color bar depicts T-statistics. The scatter plot shows the relationship between mean FA asymmetry values under the significant cluster and dorsal attention network functional laterality indices, with a least squares line superimposed in red

Lower laterality indices in the altered region of the dorsal attention network were associated with higher rightward FA asymmetry in the superior longitudinal fasciculus in the MS group (p < 0.02). Altered functional laterality of the default mode network did not correlate with the asymmetry of diffusion characteristics in the cingulum (Fig. 4).

## Discussion

In this study, we report differences of functional lateralisation in resting state networks between multiple sclerosis patients and healthy controls. The default mode network showed increased leftward functional laterality of the angular gyrus in MS patients, which was associated with better performance on the BVMT-R task. Previous studies established that the default mode network is often implicated in MS (Tahedl et al. 2018b). A leftward shift in functional lateralisation might represent an adaptation mechanism, as preserved cognitive performance is associated with leftward lateralisation of the default mode network, e.g. in MCI (Liu et al. 2018). Indeed, juvenile MS patients demonstrated increases in DMN FC when completing working memory training (Hubacher et al. 2015). In healthy participants, we observed right side dominant functional laterality in the posterior intraparietal sulcus regarding dorsal attention network functional connectivity, which is in line with previous accounts of right hemisphere dominance in attention (Lunven and Bartolomeo 2017; Shulman et al. 2009) and rightward lateralisation of attention-related functional resting state networks (Agcaoglu et al. 2015; Bartolomeo and Seidel Malkinson 2019). This right side dominancy diminished and, in some cases, shifted to left side dominancy in multiple sclerosis patients. These findings signify that functional connectivity lateralisation changes may occur regardless of satisfactory clinical status in multiple sclerosis when taking into account that patients in this study were in good condition clinically and exhibited little difference in overall intrinsic network connectivity compared to healthy controls. The shift of functional laterality from right hemisphere dominance in the dorsal attention network might be explained by a disturbance of the integration of sensory information in the posterior intraparietal sulcus as a consequence of disease pathology. The right intraparietal sulcus exhibits diminished activation in response to attention and working memory tasks in multiple sclerosis according to a recent meta-analysis (Kollndorfer et al. 2013). In our previous study we also showed that functional connectivity over and above task-related synchronization decreases between the right intraparietal sulcus and the right frontal eye field, another important region in the dorsal attention network, during a visual attention task (Veréb et al. 2020). These results are corroborated by earlier accounts of a shift of perceptual bias in multiple sclerosis patients, which, based on our findings, might be caused by a disruption of interhemispheric balance in the attention system (Gilad et al. 2006). Hemispheric asymmetry in white matter microstructure has not been extensively investigated in MS so far. There have been reports that lesions preferentially appear in the left hemisphere (Charil et al. 2003). Since asymmetry in the fractional anisotropy of major white matter tracts reportedly underlies several lateralised cognitive functions [e.g. language (Büchel et al. 2004)], it is possible that the inverse relationship between dorsal attention network functional laterality and fractional anisotropy in the superior longitudinal fasciculus represents a structural rearrangement in response to connectivity alterations. This study has several limitations. The choice of the number of components in the independent component analysis influences the spatial layout of resulting components, which means that lateralised networks can appear as separate components if the dimensionality is set high enough. Here we used a pre-set number of components used in previous studies to reproduce earlier accounts of resting state networks, which are widely accepted in the literature. Furthermore, although we made an effort to correct for the slight differences in age and biological sex distribution between the investigated groups by including these variables as nuisance regressors in the statistical model, their effect on resting state network lateralisation might present a remaining bias that has to be addressed in future studies (Table 1).

**Table 1:** Study population demographics

Demographics	RRMS (N = 24)	Healthy (N = 25)
Age (years, ± SD)	41.18 ± 8.85	37.45 ± 12.17
Sex (male/female)	6/18	9/16
Disease duration (years, ± SD)	10.38 ± 6.47	–
EDSS (median, range)	1 (0–3)	–
T2-hyperintense lesion volume (cm3, median, range, ± SD)	4 (0.52–21.51; ± 4.99)	–
Treatment	Glatiramer-acetate: 10 Teriflunomide: 6 Fingolimod: 3 Interferon B-1a: 3 Natalizumab: 1 Cladribine: 1	–

*SD* standard deviation

## Conclusions

Our results corroborate previous evidence of altered resting state network function in relapsing–remitting multiple sclerosis, and show that the disruption or adaptation of functional lateralisation patterns might contribute to cognitive dysfunction, a highly prevalent and problematic feature of the disease, even in patients with mild disability.

## Data Availability

Data is available through personal correspondence after consideration by the local ethics committee.

## Code Availability

Analyses were performed using FMRIB’s Software Library (FSL) v5.0.10, available at: https://fsl.fmrib.ox.ac.uk/fsl/
